# Retention Challenges in Opioid Use Disorder Treatment: The Role of Comorbid Psychological Conditions

**DOI:** 10.5811/westjem.38089

**Published:** 2025-07-18

**Authors:** David C. Seaberg, Jamie McKinnon, Lyn Haselton, Patrick Palmieri, Jason Kolb, Suman Vellanki, Mary Moran, J. Chika Morah, Nicholas Jouriles

**Affiliations:** * Northeast Ohio Medical University, Summa Health System, Department of Emergency Medicine, Akron, Ohio; † Northeast Ohio Medical University, Summa Health System, Department of Psychiatry, Akron, Ohio

## Abstract

**Introduction:**

Comorbid psychological conditions have an impact on opioid use disorder (OUD). We measured multiple psychological tests in OUD patients who entered an emergency department (ED)-based medication for opioid use disorder (MOUD) program to determine whether any test correlated with six-month retention in the MOUD treatment program.

**Methods:**

Patients with OUD who were enrolled in an ED-based MOUD program over a 12-month period were eligible to participate. We surveyed enrollees using nine validated tools to assess depression, anxiety, and traumatic stress within 24 hours of their ED presentation and then at one and six months. The primary outcome was program retention rates at one and six months. Secondary outcomes were levels of clinical symptoms, substance use, and quality of life.

**Results:**

Of 143 patients enrolled in the MOUD program, 64 (44.8%) participated during the 12-month study. The mean age was 33 years, with 65% male and 35% female. Baseline surveys indicated moderate symptom severity for depression and anxiety. The Post-Traumatic Stress Disorder Checklist (PCL-5) scores showed significant traumatic stress. Retention rates were 47% at one month and 25% at six months. General well-being improved from 40% at baseline to 56% at six months. Average income correlated (0.51) with six-month retention, suggesting that those with financial means were more likely to remain in treatment. The Life Events Checklist (LEC-5) correlated (0.41) with six-month retention. This indicates that the more trauma an individual experienced, the less likely the person would remain in treatment.

**Conclusion:**

Higher income and lower post-traumatic stress disorder scores had higher retention rates in a medication-based opioid use disorder program. Psychological surveys of patients entering a MOUD program may help predict treatment retention. There will likely be challenges in keeping patients with extensive trauma histories retained in treatment.

## INTRODUCTION

Patients with opioid use disorder (OUD) often have significant comorbid psychological health concerns, including depression, anxiety, and post-traumatic stress disorder (PTSD).^1^–^4^ The extent to which these psychological comorbidities affect participation in a medication for opioid use disorder (MOUD) treatment program is unknown.^5^ Longitudinal evaluation of mental health and well-being in patients receiving MOUDs alone, without counseling, is lacking in the scientific literature.^6^ Two studies have shown that patients taking buprenorphine experience decreases in psychiatric symptoms (depression and anxiety) in the first three months of treatment.^5^,^7^ Another study with longer follow-up found that mental health (as measured by the Short Form Health Survey-36) significantly improved from baseline to 12 months in patients enrolled in a study of extended-release buprenorphine injections.^8^ All these studies demonstrated that the most significant improvement in mental health symptoms occurred in the first month of treatment. More research is needed to understand changes in mental health and well-being in patients receiving MOUD in a primary care setting.

We measured multiple validated and reliable psychological surveys, including the Personal Health Questionnaire (PHQ-8) measuring depression symptoms,^9^ Generalized Anxiety Disorder 7-item scale (GAD-7) to measure anxiety symptoms,^10^ the PTSD checklist (PCL-5) to measure PTSD symptoms,^11^ World Health Organization well-being index (WHO-5) to measure well-being,^12^ Brief Addiction Monitor (BAM-R) to measure addiction symptoms,^13^ Adverse Childhood Experiences Survey (ACE) to measure adverse childhood trauma,^14^ life events checklist (LEC-5) to measure post-tramatic stress,^15^ and the Protocol for Responding to and Assessing Patients’ Assets, Risks, and Experiences scale (PRAPARE) to measure social determinants of health^16^ in patients entering an ED-based MOUD program to ascertain predictors of treatment retention. These surveys measure a cumulative score that correlates with symptoms and can be followed over time. The primary outcome was retention rates in the program at one and six months. Secondary outcomes were levels of various clinical symptoms (eg, depression, anxiety), substance use, and quality of life. We correlated individual survey findings with retention in a MOUD program measured as seeing an addiction medicine clinician at one and six months.

## METHODS

### Participants

All patients who enrolled in any of our four ED-based MOUD programs during the 12-month study period were eligible to participate in this study. The vast majority of these patients presented to the ED asking for treatment for their OUD. The remainder were enrolled into the MOUD program through our screening, brief intervention, and referral to treatment (SBIRT)^17^ questioning required in our nurse triage process. Patients electing inpatient OUD treatment were not eligible. The patients enrolled were followed up at one and six months; therefore, the study was conducted over 18 months.

Our MOUD program comprises one urban hospital-based ED, one community hospital-based ED, and two suburban freestanding EDs We sought to compare the following outcomes for our MOUD enrollees:

- Program retention, defined as attendance at follow-up appointments (in-person or telehealth) or patient contact via telephone at one and six months after the initial ED MOUD encounter; this was measured through chart review.
- Well-being and quality of life.^18^ These were assessed at baseline, and at one and six months post initial MOUD encounter.

**Population Health Research Capsule**

What do we already know about this issue?

*Use of baseline psychological tests in patients with opioid use disorder (OUD) to predict retention in an ED-based treatment program has never been studied*.

What was the research question?

*Does baseline psychological testing in patients in an ED-based medication for OUD (MOUD) program correlate with six-month retention?*

What was the major finding of the study?

*Patients with higher income and lower PTSD scores had higher retention rates in a MOUD treatment program at six months (P < .05)*.

How does this improve population health?

*Opioid use disorder is a significant health concern. Trauma-informed surveys may help predict who will remain in MOUD treatment*.

We used previously validated and reliable measures such as the PHQ-8, GAD-7, PCL-5, WHO-5, Difficulties in Emotion Regulation Scale (DERS), BAM-R, ACE, LEC-5, and PRAPARE. This allowed us to track participant outcomes efficiently, compare with other systems, and to replicate our program at different sites. Surveys monitored and evaluated depression, anxiety, PTSD symptoms, quality of life, substance use and risk, and social determinants of health, including lifetime trauma and adversity.

The addiction care coordinator (ACC) assisted all patients in completing the surveys, in person or remotely, at the applicable time points, and aggregated all relevant data for analysis. If a patient entered the MOUD program at night, the ACC would follow up with the patient the next day, enroll the patient in the study, and complete surveys by phone. Surveys could also be completed through an online survey program (Neuroflow [NeuroFlow, Inc, Philadelphia, PA] or RedCap [Research Electronic Data Capture hosted at Northeast Ohio Medical University. The results provided information to assess changes over time, evaluate differences between patients served at different sites, and improve our program, leading to better patient outcomes, program sustainability, and a healthier community. The chart abstractors for collecting survey data and retention rates were blinded to the study hypothesis.

### Program Description and Evaluation Focus

#### Measures

We administered the mobile health (mHealth) app usability questionnaire (MAUQ),^19^ which has versions for patients and healthcare professionals. The MAUQ provides information about three domains: ease of use; usefulness, interface; and satisfaction. We used two platforms (NeuroFlow and REDCap) to assess telehealth’s efficacy in a MOUD program.

### Emotion Dysregulation

Given that substance use is one way that people cope with emotional distress, emotion (dys)regulation is an essential psychological process to measure for assessing the risk of substance use/relapse and identifying potential targets of intervention that ultimately may help improve engagement in substance use treatment and outcomes. The DERS^20^ assesses various aspects of emotion dysregulation, including non-acceptance of emotional responses, difficulties engaging in goal-directed behavior, impulse control difficulties, lack of emotional awareness, limited access to emotion regulation strategies, and lack of emotional clarity. This tool can be especially useful in helping patients identify areas for growth in how they respond to their emotions, especially those with borderline personality disorder, generalized anxiety disorder, or substance use disorder.

### Trauma/Adversity and Social Determinants of Health

The US Centers for Disease Control and Prevention define social determinants of health as the conditions in which people live, learn, work, and play that affect various health risks and outcomes.^21^,^22^ They include demographic and psychosocial factors such as race, ethnicity, education, employment, income, language barriers, food insecurity, housing stability, neighborhood/environmental factors, and more.^23^ The PRAPARE is a standardized patient social risk assessment tool.^16^ Social determinants of health are correlated with substance use and accessing treatment services. Exposure to traumatic events or other types of adversity is also known to be associated with increased risk of substance use as well as to the likelihood of treatment seeking, engagement, and outcomes. The LEC-5^15^ assesses 17 categories of traumatic events (eg, physical assault, natural disasters, combat) and whether someone, in adulthood or childhood, has ever experienced, witnessed, or learned about these events happening to a loved one. Adverse trauma events were not weighted for severity when using the LEC-5.

The ACE^14^ questionnaire includes some of these trauma types as well as other types of adversity and social determinants of health, such as neglect and family dysfunction, which are also associated with substance use and other health risks and outcomes. Together, the PRAPARE, ACE, and LEC-5 provide a thorough understanding of psychosocial factors and experiences across the lifespan that are associated with substance use, treatment, and outcomes.

### Mental Health Symptoms and Quality of Life

We included several clinical symptom-severity measures in the data collection plan. The PHQ-8, GAD-7, and PCL-5 assess symptoms of depression (excluding suicidality), anxiety, and traumatic stress, respectively. It is well established that these symptom types highly co-occur with substance use. Thus, these measures serve as predictors of treatment engagement/retention and secondary outcomes of their own. In addition, because it is vital to assess broader outcomes than just clinical symptoms, we administered the WHO-5 to evaluate quality of life.

### Substance Use, Mental Health Symptoms, and Quality of Life

The BAM-R is designed to measure progress in patients who are in treatment for substance use disorder. It assesses alcohol and drug use, as well as risk and protective factors associated with use or sobriety, within the prior 30 days. Several clinical symptom-severity measures are also included in the data collection plan. The PHQ-8, GAD-7, and PCL-5 assess symptoms of depression (excluding suicidality), anxiety, and traumatic stress, respectively. It is well established that these symptom types highly co-occur with substance use

## ANALYSIS AND INTERPRETATION

The primary outcome was retention in the program at one and six months post-initial ED MOUD encounter. Secondary outcomes were severity levels of various clinical symptoms (eg, depression, anxiety). We obtained descriptive statistics for all demographic, clinical, and other variables (predictors and outcomes) in the measurement plan. A correlation table was generated to examine associations among the variables. We conducted chi-square tests for categorical variables (count and percent variables), and we performed *t*-tests and analysis of variance for continuous variables, with *P* < .05 considered statistically significant. We used regression analyses to identify significant predictors of retention. This analysis included covariates/control variables (eg, social determinants of health) associated with the outcome variable. We assessed clinical symptom-severity changes within groups over time with paired *t*-tests using baseline and final endpoint measurements. Since this was part of a QI project, the Summa Health System Institutional Review Board deemed it to be patients’ assets, risks, and experiences scale (PRAPARE) exempt from review.

## RESULTS

During the one-year study period (September 2021–August 2022), our EDs saw 148,251 patients, with a total of 2,875 OUD-confirmed patients. Of confirmed OUD patients, 143 enrolled in our ED-based MOUD programs. The vast majority (96%) of those enrolled presented to the ED for treatment for their OUD. The rest (4%) were identified with an OUD and offered MOUD through the triage-nurse SBIRT process. All patients enrolled had cell phone access to the internet to complete surveys. Of the enrolled patients, 64 (45%) completed a baseline assessment. The patient’s mean age was 33 years (SD 8), ranging from 20–62 years. Demographics were as follows: 67% male; 70% single; 17% married; and 13% divorced/separated/widowed. The racial profile noted 81% White, 9% Black, 3% American Indian/Alaskan, and 7% identified as Hispanic or Latino. Regarding their living situation, 30% reported living alone, and 75% reported having housing; however, 41% reported concerns about losing their housing. In total, 86% of patients had a high school diploma, and 60% were unemployed. The average income was $25,000 annually, and 71% of the population had Medicaid health insurance.

For the primary outcome measure, surveyed patients had a retention rate in the MOUD program of 47% (95% confidence interval [CI] 46.0–48.0; 30 patients) at one month and 25% (95% CI 24.1–25.9; 16 patients) at six months. The Consolidated Standards of Reporting Trials flow diagram is noted in the Figure.

### Baseline Clinical Symptom Levels

Patients reported scores that indicated moderate symptom severity for both depressive symptoms (PHQ-8) and anxiety (GAD-7). The PCL-5 scores showed significant traumatic stress levels that approached the cutoff of 33 for indicating probable PTSD. The WHO-5 scores were <13, indicating poor general well-being. Scale scoring for the BAM-R, considered very preliminary regarding its psychometric properties, includes use, risk factor, and protective factor scores. All three scales exceeded cutoffs that warranted further examination or clinical attention (Table 1).

### Follow-Up Clinical Symptom Levels

We documented several areas of improvement at the six-month follow-up. Both depressive and anxiety symptoms improved at the six-month follow-up to <10 and <8, respectively, indicating an improvement from moderate to mild symptoms (*P*<.05). General well-being improved from 40% at baseline, a score of 9.91, to a score of 14.11, or 56%, at the six-month follow-up, which exceeds the suggested cutoff of at least a 10% improvement in percentage score to indicate improved well-being. The BAM-use score decreased below the clinical cutoff of one at one month and was above one at six months. Furthermore, the average BAM-risk factor score decreased below the clinical cutoff 12 at one and six months. The average BAM-protective score was correct at the clinical cutoff at one and six months. Another way to interpret the BAM is to compare the BAM-risk and BAM-protective scores to see which is higher. While the risk score was somewhat higher than the protective score at baseline (as expected), the protective score was slightly higher than the risk score at one and six months, which suggests the patients were somewhat less at risk of using opioids.

### Exposure to Trauma and Adversity

The data indicate this patient population has experienced significant amounts of trauma and adversity. The ACE questionnaire assesses 10 different types of adverse childhood experiences in the areas of abuse, neglect, and family dysfunction. It returns a score from 0–10, indicating the number of types (not frequency) of experiences witnessed, learned of, or experienced by 18 years of age. Patients averaged four types of adverse childhood experiences. The ACE score was not statistically different at baseline for patients retained at six months compared to those not retained at six months.

The LEC-5 assesses 17 types of traumatic experiences in childhood and/or adulthood. For the patients who completed this questionnaire, trauma was highly prevalent. Patients, on average, reported directly experiencing four different types of traumas, witnessing two types of traumas, and learning about two types of traumas happening to loved ones. (As with the ACE, the LEC does not assess the number of experiences of these different types.) The LEC-5 was lower for the patients who were retained in the program at six months, 5.88 vs 2.33, noting clinical and statistical significance (*P* < .05). The LEC-Happened (number of types of trauma directly experienced by the person) correlated −0.41 (95% CI 0.39, −0.43) with six-month retention. This suggests that the more types of trauma experienced throughout life, the less likely the person is to remain in treatment at six months.

One other statistical and clinical correlation was noted in the survey. The PRAPARE14 (average annual income) correlated 0.51 (95% CI 0.49 – 0.53) with six-month retention, suggesting that people with relatively more financial means were more likely to remain in treatment at six months.

### Emotion Regulation

The DERS measures emotion regulation problems. Baseline data for patients showed an average score of 81.91 (95% CI 71.9 – 91.9), which was not significantly different from the six-month score of 79.77 (95% CI 76.4 – 83.1, *P* > .05). This was similar to the scores in a published sample of adults who presented at a general outpatient clinic and were diagnosed with one or more disorders in the *Diagnostic and Statistical Manual of Mental Disorders, 5^th^ Ed*.^24^ The sample size was again low, but here are the findings:

- The DERS score at baseline was statistically and clinically correlated with the baseline BAM score (0.75 (95% CI 0.71 – 0.79)).
- The DERS score at baseline was statistically and clinically correlated with baseline GAD-7 score, (0.62 (95% CI 0.58 – 0.66)).
- The DERS score at baseline was statistically and clinically correlated with the baseline WHO-5 score (−0.73 (95% CI −0.69, −0.77).

Thus, more difficulties in emotion regulation at baseline are associated with more risk of use/relapse, more generalized anxiety, and lower general well-being. This underscores that emotion regulation may be a significant factor to consider in MOUD treatment and that bolstering emotion regulation skills may be an important focus of treatment.

Table 2 lists additional factors from the social determinants of health. Almost half of the sample reported seeing or talking to people they cared about and felt close to no more than twice a week. Additionally, 80% reported feeling quite a bit or very much “stressed,” and 31% had spent more than two consecutive nights in jail/prison/detention/juvenile correction in the prior year. A total of 5% identified as refugees. Surprisingly, 95% reported feeling physically and emotionally safe where they lived. However, 15% reported being afraid of a partner or ex-partner sometime in the previous year. We assessed the patient satisfaction level of 39 individuals at the end of the first visit (95% CI 93.9 – 96.1) who reported being “satisfied” or “very satisfied” with their care experience in the MOUD program.

## DISCUSSION

This project examined using previously validated well-being and psychosocial surveys on MOUD program retention rates. The surveys were administered by the ACCs during the first MOUD visit and subsequently by use of the NeuroFlow remote patient monitoring platform. The results of our surveys indicate several significant trends and correlations. First, the LEC-5 (lifetime trauma exposure) scores significantly correlated with MOUD program retention. Specifically, the LEC-happened (number of types of trauma directly experienced by the person) correlated −0.41 with six-month retention. This suggests that the more types of trauma someone experiences throughout their life, the less likely the person would remain in treatment at six months. An epidemiological study with nationally representative samples reported that 50–60% of the population will experience at least one traumatic event in their lives, and a third will experience three or more instances of trauma.^25^ Survey participants who were not retained in the MOUD program at six months averaged 5.88 on the LEC-5 at baseline, and people who were maintained at six months averaged 2.33 on the LEC-5. These are significantly different (*P* = .02). This further underscores that the more types of trauma (not specific to childhood) someone experiences is an essential factor in whether they remain in MOUD treatment at six months. Thus, if MOUD treatment is not trauma-informed, there likely will be more difficulty keeping patients with extensive trauma histories retained in treatment.

The literature shows that higher ACE scores are associated with a higher risk of numerous adverse health, academic/occupational, interpersonal, and behavioral outcomes, including substance use.^26^ Scores of ≥4 are consistently associated with high rates of these adverse outcomes. Half of the respondents had scores of ≥4, consistent with a study reporting that with each additional point on the ACE questionnaire, there is a 17% increase in the risk of relapse during MOUD treatment, usually after the first visit.^27^ The high ACE scores in our patients highlight the significant relapse risk related to childhood trauma history. Fortunately, in that study,^26^ each treatment visit was associated with a 2% reduction in the risk of relapse. This highlights the importance of engaging patients and retaining them in treatment.

The DERS score correlated with the BAM-R risk score, GAD-7 score, and WHO-5 score in our survey patients. Thus, more difficulties in emotion regulation at baseline are associated with more risk of use/relapse at baseline, more generalized anxiety at baseline, and lower general well-being at baseline. This underscores that emotion regulation may be a significant factor to consider in MOUD treatment and that bolstering emotion regulation skills may be an important focus of treatment. PRAPARE14 (average annual income) was correlated 0.51 with six-month retention. This suggests that people with relatively more financial means were more likely to remain in treatment for six months.

Although the sample size was small and the findings must be considered preliminary, there is evidence that patients showed improvement in depression, anxiety, PTSD symptoms, and general well-being during the initial months of MOUD treatment. This demonstrates our MOUD program’s positive effect in improving physical and mental health in patients with OUD. Overall, 95% of our patients were satisfied or very satisfied with what they received at their initial visit.

Finally, the surveys gave us a better understanding of the social determinants of health that our OUD patients experience. We believe that measuring the survey results over time, specifically using the PRAPARE survey, allowed us to examine and understand the social dieterminants of health that our OUD patients experienced over time. Understanding this is important to meeting the social needs of this population as outlined in the Health Opportunity and Equity Initiative and State Health Improvement Plan. Not only is the stigma surrounding OUD a barrier to effective treatment, but psychosocial issues of transportation, food, housing, and finances all affect patients’ ability to remain in a coordinated program such as a MOUD.

## LIMITATIONS

The small sample size limited this study. This was directly related to the number of surveys performed and the difficulty in getting this patient population to work with traditional medicine. We used the Neuroflow program to administer surveys online, but the response rate was low. The use of mhealth apps could considerably assist both individuals and healthcare professionals in the prevention and management of chronic diseases, in a person-centered manner. However, there is a lack of both standardized methods to measure the clinical outcomes of mHealth apps and techniques to encourage user engagement and behavior changes in the long term. Current research is exploring ways to overcome these barriers^28^

We attempted to improve the response rate by providing gift cards for continued participation, but this did not increase the response rate. We also educated our follow-up behavioral health professionals on the importance of this study, and to assist us in procuring survey responses. There was a decrease in the number of patients entering our MOUD program compared to previous years, which may have contributed to the small sample size. The reasons for this are unclear, but several factors could be involved. First, the primary opioid used in our area is fentanyl, and some have postulated that MOUD may be less effective due to the increased potency of fentanyl. Additionally, fentanyl may lead to worsening withdrawal symptoms, making MOUD treatment less palatable and leading to an increase in inpatient admission for OUD. Because we have an extensive inpatient detox program, many people opted for inpatient therapy. The expansion of MOUD programs at other sites in our community may have reduced the number of patients in our MOUD program. Lastly, there is debate regarding the effectiveness of behavior counseling on retention in MOUD patients. Of the three studies that examined this question, only one found that behavioral counseling improved retention rates.^29^–^31^

## CONCLUSION

Psychological surveys of patients with opioid use disorder entering a medication for OUD program may help predict treatment retention. Our study found patients with higher income and lower PTSD scores had higher MOUD retention rates at six months. If MOUD treatment is not trauma-informed, there will likely be challenges in keeping patients with extensive trauma histories retained in treatment. Emergency department staff may need to examine the potential for PTSD and whether it affects retention in MOUD programs.

## Supplementary Information



## Figures and Tables

**Figure:**
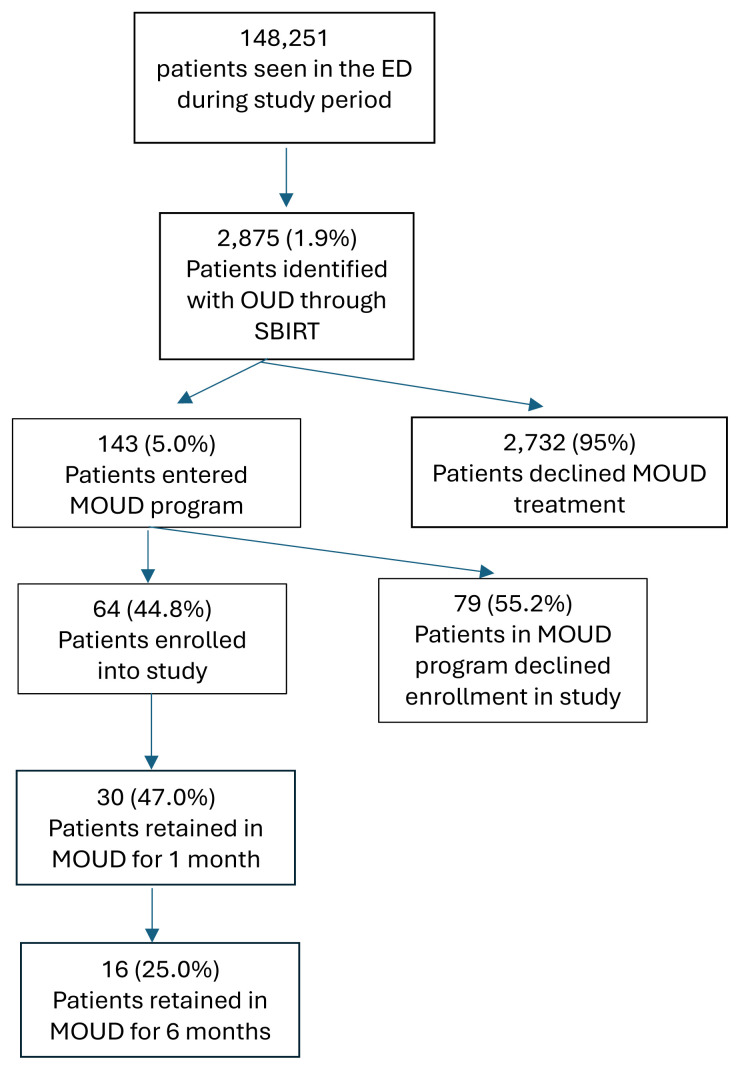
Consolidated Standards of Reporting Trials (CONSORT) flowchart for patient enrollment. *ED*, Emergency Department; *OUD*, Opioid use disorder; *MOUD*, Medication for opioid use disorder; *SBIRT*, Screening, brief intervention

**Table 1:** Baseline clinical symptom levels.

Construct and measure	Baseline (n=30) Average score (SD)	6 months (n=16) Average score	Baseline symptom severity
Depressive Symptoms (PHQ-8)	13.80 (6.78)	9.66 (4.78)*	Moderate > 10 = Major > 20 Severe
General Anxiety (GAD-7)	10.88 (6.52)	7.78 (4.33)*	Moderate 10–14 = Moderate > 15 = Severe
Traumatic Stress (PCL-5)	28.77 (19.29)	19.86 (13.22)*	Significant/High > 30 = Severe
PTSD (LEC-5)	5.88 (2.52)	2.33 (1.63)*	Significant PTSD > 5 = Severe
General Well-being (WHO-5)	9.91 (6.35)	14.11 (5.89)*	Poor well-being < 10 = Poor
Addiction (Use BAM-R)	3.10 (2.33)	1.81 (1.72)	Cutoff of 1+ correlates with high addiction
Childhood Trauma (ACE)	4.15 (3.15)	3.92 (2.89)	Moderate > 4 = Significant
Emotional Dysregulation (DERS)	81.91 (13.84)	79.77 (13.66)	High > 82 = Severe

* p < .05.

*PHQ-8*, Personal Health Questionnaire; *GAD-7*, General Anxiety Disorder; *PCL-5*, Post-Traumatic Stress Disorder Checklist; *PTSD*, post-traumatic stress disorder; *LEC-5*, Life Events Checklist; *WHO-5*, World Health Organization well-being index; *BAM-R*, Brief Addiction Monitor; *ACE*, Adverse Childhood Experiences survey; *DERS*, Difficulties in Emotional Regulation Scale.

**Table 2:** Social determinants of health in the previous year.

Social Determinants of Health Factor	Percentage of sample n = 64
Patients or family members had been unable to get food when needed	30%
Patients or family members had been unable to get clothing when needed	30%
Patients or family members had failed to get utilities when needed	32%
Patients or family members had been unable to get childcare when needed	6%
Patients or family members had failed to get health care when needed	23%
Patients or family members had failed to get a phone when needed	21%
Lack of transportation kept them from medical appointments or medication	43%
Lack of transportation kept them from other appointments, work, or getting things they needed	9%
