# Supplementary figures and images for: Retention Challenges in Opioid Use Disorder Treatment: The Role of Comorbid Psychological Conditions
Source: West J Emerg Med. 2025 Jul 18;26(4):897–904. doi: 10.5811/westjem.38089 (PMC12342412; doi:10.5811/westjem.38089)

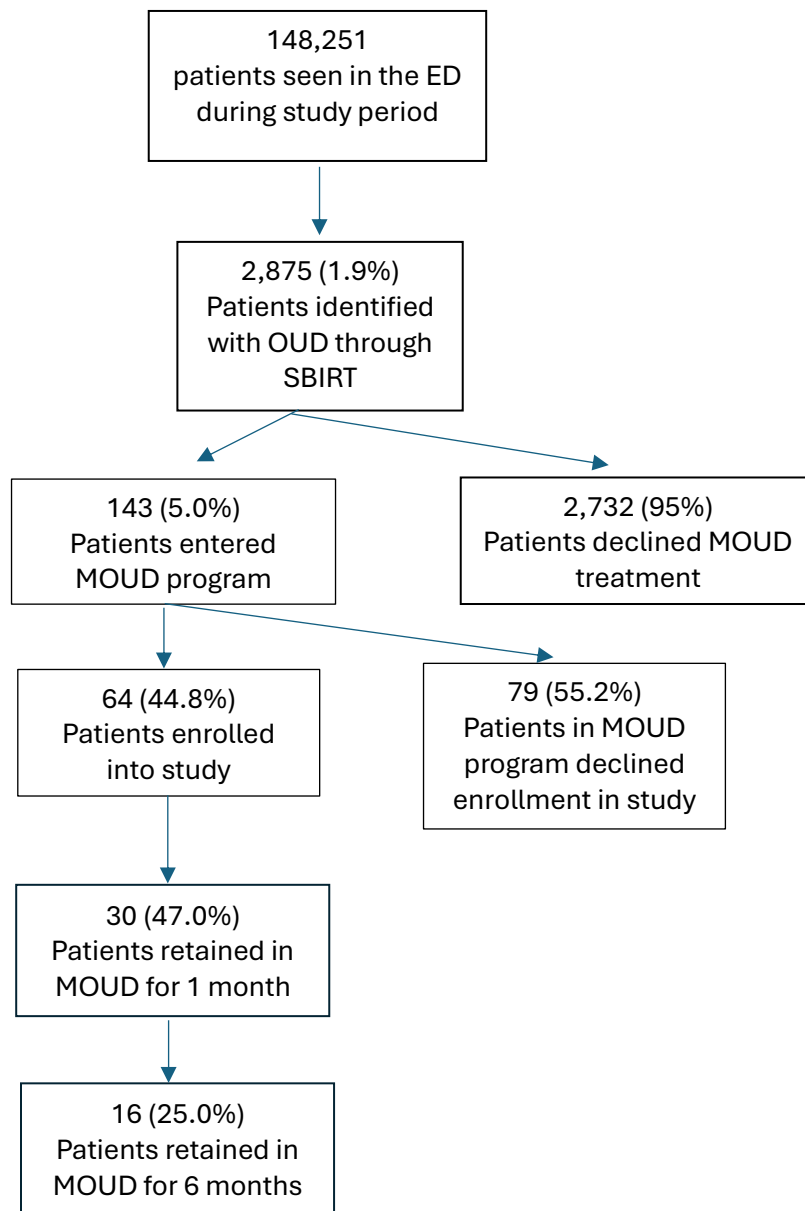

Supplement: Supplementary file 1 [file wjem-26-897-g001.pdf]
